# Genomic Markers Associated with Cytomegalovirus DNAemia in Kidney Transplant Recipients

**DOI:** 10.3390/v15112227

**Published:** 2023-11-08

**Authors:** Guy Shapira, Hadas Volkov, Itai Fabian, David W. Mohr, Maria Bettinotti, Noam Shomron, Robin K. Avery, Ravit Arav-Boger

**Affiliations:** 1 Faculty of Medicine, Tel Aviv University, Tel Aviv 69978, Israel; guyspersonal@gmail.com (G.S.);; 2 Edmond J. Safra Center for Bioinformatics, Tel Aviv University, Tel Aviv 69978, Israel; 3 Johns Hopkins Genetic Resources Core Facility, Johns Hopkins University School of Medicine, Baltimore, MD 21287, USA; 4 Immunogenetics Laboratory, Johns Hopkins University School of Medicine, Baltimore, MD 21287, USA; mbettinotti@jhmi.edu; 5 Department of Medicine, Division of Infectious Diseases, Johns Hopkins University School of Medicine, Baltimore, MD 21287, USA; ravery4@jhmi.edu; 6 Department of Pediatrics, Division of Infectious Diseases, Medical College of Wisconsin, Milwaukee, WI 53226, USA

**Keywords:** human cytomegalovirus, kidney transplantation, genetic susceptibility, whole-exome sequencing, CMV DNAemia

## Abstract

Human cytomegalovirus (CMV) is a major pathogen after solid organ transplantation, leading to high morbidity and mortality. Transplantation from a CMV-seropositive donor to a CMV-seronegative recipient (D+/R−) is associated with high risk of CMV disease. However, that risk is not uniform, suggesting a role for host factors in immune control of CMV. To identify host genetic factors that control CMV DNAemia post transplantation, we performed a whole-exome association study in two cohorts of D+/R− kidney transplant recipients. Quantitative CMV DNA was measured for at least one year following transplantation. Several CMV-protective single-nucleotide polymorphisms (SNPs) were identified in the first cohort (72 patients) but were not reproducible in the second cohort (126 patients). A meta-analysis of both cohorts revealed several SNPs that were significantly associated with protection from CMV DNAemia. The copy number variation of several genes was significantly different between recipients with and without CMV DNAemia. Amongst patients with CMV DNAemia in the second cohort, several variants of interest (*p* < 5 × 10^−5^), the most common of which was NLRC5, were associated with peak viral load. We provide new predictive genetic markers for protection of CMV DNAemia. These markers should be validated in larger cohorts.

## 1. Introduction

Infection with human cytomegalovirus (CMV), a member of the herpesvirus family, is common in humans. Seroprevalence rates increase with age, reaching 90% in individuals older than 80 years [1]. CMV establishes lifelong persistent infection, and individuals typically remain asymptomatic. In immunocompromised hosts, CMV causes significant morbidity and mortality [2,3,4,5]. Over 75% of solid organ transplant recipients are newly infected or reactivate latent CMV after transplantation. Kidneys are the most commonly transplanted solid organs, and CMV-seronegative recipients from a CMV-seropositive donor (D+/R−) are the subgroup at highest risk for CMV infection and disease [6]. Without prophylaxis, infection is diagnosed in 50–60% of kidney transplant recipients [7]. Prophylactic antiviral therapy has decreased the incidence of CMV infection and disease in the early post-transplant period, but these agents have significant toxicities [8]. Moreover, late-onset CMV disease is associated with allograft failure and mortality [9].

The CMV double-stranded DNA genome is around 235 kbp and has the largest genome among herpesviruses. Multiple CMV-encoded gene products are devoted to host immune evasion, among which are chemokines, chemokine receptors, and cytokines, allowing CMV to modify and interfere with host immune responses [10,11,12,13]. The role of host immune response in CMV reactivation following organ transplantation has been difficult to study, partially because of sample availability, the complexity of CMV interaction with multiple cellular pathways, and the ability to identify a defined outcome measure.

CMV infection following organ transplantation was associated with variants in several human genes: those encoding toll-like receptors (TLRs) [14], programmed death-1 (PD-1), and interleukin-12p40 (IL12B). Polymorphisms in the interferon lambda 3/4 (IFNL3/4) region also influenced susceptibility to CMV replication in solid organ transplant recipients. However, no association was found between ten genetic variants in TLR4, TNF-α, IL10, IFN-γ, and IL37 in CMV-positive renal allograft recipients and active infection in a subanalysis (116 blood samples) of a prospective randomized VIPP study (NCT00372229) [15], demonstrating the limitation of the small cohorts and the need for additional genetic epidemiological studies with large cohorts to elucidate the genetic mechanisms of CMV infection and reactivation following solid organ transplantation.

A one-year retrospective study of CMV reactivation (based on CMV antigenemia) in 200 kidney transplant recipients investigated the relationship between CMV infection and 59 HLA alleles. Recipients with HLA-B44 were more commonly infected with CMV compared with patients without this allele (*p* = 0.024). In contrast, recipients with HLA-DR1 were less likely to have CMV reactivation than patients without this allele (31% vs. 55%, respectively, *p* = 0.02) [16]. A positive correlation between the presence of the HLA-E*01:03 allele in living-donor kidney recipients and CMV reactivation during the first year after transplantation was reported, suggesting that HLA-E genotyping may help identify CMV replication-prone patients [17].

The copy number variation (CNV) is another genomic variation, which may play an important role in the susceptibility to infectious diseases [18]. For example, a relationship between CCL3L1 dose and susceptibility to HIV/AIDS was reported. Possession of a CCL3L1 copy number lower than the population average was associated with markedly enhanced susceptibility to HIV [19]. CNV was reported in CMV reactivation. A significant association was found between donor NKG2C copy number and protection against CMV reactivation after double cord blood transplantation [20].

Here, we aimed to investigate genetic markers of CMV DNAemia in two cohorts of kidney transplant recipients with a well-defined phenotype. The recipients were all CMV-seronegative at the time of transplantation, and their donors were all CMV-seropositive.

## 2. Materials and Methods

### 2.1. Patients and Samples

Serum samples were collected from kidney transplant recipients and stored in the Immunogenetics Laboratory at Johns Hopkins (Baltimore, MD, USA), under IRB approval. CMV infection was monitored for at least one year after transplantation. Data collected from recipients of a kidney transplant included: patient age at transplant, gender, race, any positive CMV PCR (date and viral load), peak viral load, immunosuppression therapy, and CMV prophylaxis.

### 2.2. Sequencing and Genotyping

Samples were sequenced and genotyped at the Johns Hopkins University Genetic Resources Core Facility DNA Services (Baltimore, MD, USA). Illumina InfiniumQCArray-24v1-0 array (Illumina, San Diego, CA, USA) was used to confirm gender and assess relatedness. Exome capture was performed using Agilent SureSelectXT HumanAllExon (V6 S07604514) (Agilent, Santa Clara, CA, USA) for cohort A and Twist Human Core Exome (Twist Bioscience, South San Francisco, CA, USA) for cohort B. Cohort A was sequenced using the HiSeq2500 platform (Illumina, San Diego, CA, USA), 125 bp paired-end reads. Cohort B was sequenced using the NovaSeq 6000 platform (Illumina, San Diego, CA, USA), with 100 bp paired-end reads. Raw sequencing data were aligned by BWA-Mem v0.7.15 [21] to the GRCh37 genome reference. Variant calling was performed according to the Genome Analysis Toolkit (GATK v4.0.1.1) best practice, including duplicate marking, base quality and score recalibration, and joint genotyping [22]. The called variants are summarized in Supplementary File S1. Filtered variant call sets were imputed using 1000 genomes phase 3 genomic data. A full description of the process can be found in the Supplementary Data. Plink was used to aggregate genotypes and to verify sex assignment (In addition to the prior verification, detailed in the Supplementary Data), and PCA (http://pngu.mgh.harvard.edu/purcell/plink/; accessed on 7 March 2023).

### 2.3. Association and Meta-Association Analysis

Association analyses were performed for each cohort using a generalized linear model under Gemma [23]. Because of the small sample size, mixed modeling and more complex approaches were not used. Outlier samples were identified using PCA and removed prior to downstream analysis. Sex and the first principle component were used as covariates (See Supplementary Figures S1 and S2 for PCA plots). Meta-association was performed using METAL, operating on results from per-cohort tests, weighted by the size of the cohort. Because of the small number of non-Caucasian participants, a separate Caucasian-only meta-analysis was performed. Variant annotations were obtained from snpEff version 5.2 [24] and processed using in-house scripts. Allele frequencies (AF) were obtained from large-scale databases, including NCBI’s ALFA and gnomAD.

### 2.4. Copy Number Variant Analysis

Copy number variants were called using CNVKit, with the target coordinates for the capture kit used for sequencing each cohort (7485 segments in total). Each segment was matched with overlapping genes and counted only if one or more copies were gained or lost. The significance was measured by the uniformity of copy number alteration, using binomial testing with false discovery rate (FDR)-adjusted *p*-values.

### 2.5. Extra Visualization and Downstream Analysis

Locus zoom was used to visualize results loci [25]. Global Manhattan plots and the rest of the figures presented in this paper were generated using the ggplot2 package in R. Gene set enrichment was performed using hypergeometric tests, with FDR correction applied to each set family (KEGG, GO, etc.).

## 3. Results

### 3.1. Study Population

Exome variant association analysis was performed on two cohorts. Cohort A included 72 patients who received a kidney transplant between 2013 and 2016, and cohort B—126 patients who received a kidney transplant between 2005 and 2012 (Table 1) at Johns Hopkins University School of Medicine. All patients were CMV-seronegative with CMV-seropositive donors at the time of transplant. The majority of participants were Caucasians, with 16 African, one Asian, and one Hispanic in cohort A. The non-Caucasian portion of cohort A was too small to uncover reliable ethnicity-specific associations; we therefore generated an additional Caucasian-only analysis. Sex and the first principal component were used as covariates, to account for ancestry differences. The monitoring protocol for D+/R− kidney transplant recipients after completion of valganciclovir prophylaxis was CMV PCR every two weeks for three months, then CMV PCR monthly x three months. Usually, this corresponded to Months 7–9 and Months 10–12 post-transplant.

### 3.2. Induction and Maintenance Immunosuppression Regimen

Of the 198 patients, all received standard induction immunosuppression including thymoglobulin, except for 18 patients who received basiliximab and 4—daclizumab. Three patients received no induction. In addition to thymoglobulin, 38 patients also received plasmapheresis +/− rituximab. Of those, 24 were in the no-CMV DNAemia group and 14 in the CMV DNAemia group. All patients received maintenance immunosuppression with prednisone/tacrolimus/mycophenolate, except for 10 patients who received a sirolimus-based regimen (six in the non-CMV DNAemia group, four in the CMV DNAemia) and five patients who received an everolimus-based regimen (four in the no-CMV DNAemia and one in the CMV DNAemia group). There were eight patients who received belatacept, six—cyclosporine, six—alemtuzumab, one—eculizumab, and one—daclizumab for maintenance. All 72 patients in cohort A and 126 patients in cohort B received six months of valganciclovir prophylaxis at 900 mg daily (or adjusted for renal function).

### 3.3. CMV DNAemia Variant Association Analysis

**Figure 1:**
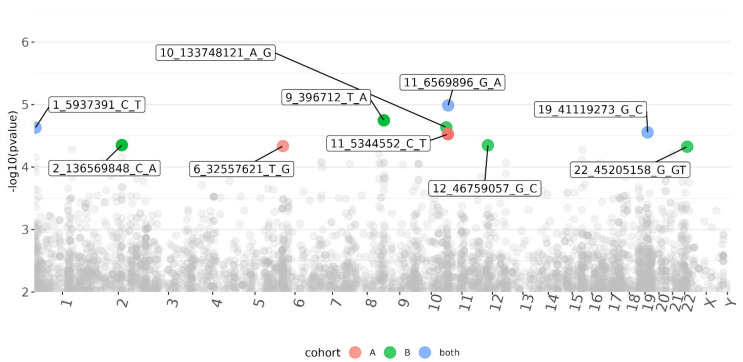
Manhattan plot of variants associated with CMV DNAemia in both cohorts of kidney transplant recipients. Each dot represents a variant, placed according to its genomic position (*x*-axis, labeled chromosome name) and the significance of the association (*y*-axis; -log10 (*p*-value); higher indicates higher significance). Associations that passed the significance threshold (*p* < 5 × 10^−5^) are labeled and colored (see Supplementary Methods S1 and S2).

**Figure 2:**
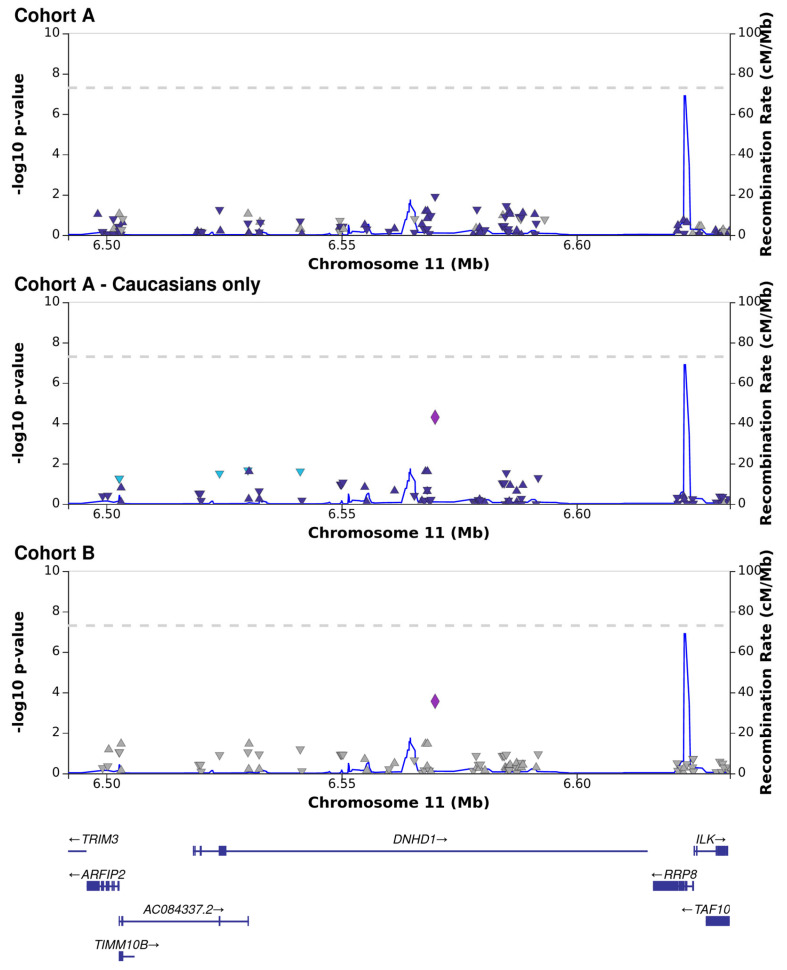
Association analysis between CMV DNAemia and genetic variants in the dynein heavy chain domain 1 (DNHD1) locus, presented for cohort A, Caucasians only in cohort A, and cohort B. Each shape represents a single genetic variant, placed on the *X*−axis according to its genomic position in relation to genes (visualized on the bottom panel) and on the *Y*−axis (left side) according to the log−scaled *p*−value of association (each of the three top panels applies to a single cohort). The recombination rate is denoted by the thin blue line and the values on the *Y*-axis (right side). Leading SNP results are marked by larger symbols, surrounding SNPs are colored according to linkage.

Association analysis was performed for each cohort independently, followed by meta-analysis. The reproducibility between the cohorts was generally low, with only a few single-nucleotide polymorphisms (SNPs) associated with the presence or absence of CMV DNAemia in the meta-analysis (Figure 1; Supplementary Files S2–S4). The results of the exome sequencing consisted of very common variants (minor allele frequency, MAF > 10%) with low linkage disequilibrium (LD). We defined a *p* < 5 × 10^−5^ as the threshold for variants of interest. Although genome-wide association studies use strict criteria for significance, it is necessary to account for the lower number of variants called from our targeted sequencing [26]. Of the five variants that passed the threshold in the meta-analysis, three were based on results from both cohorts, and an additional two were sequenced in only one of the cohorts, one from each (Table 2). All five variants are common in the general population (MAF > 10%; gnomAD). The most significant variant was a frameshift and missense mutation of Dynein Heavy-Chain Domain 1 (DNHD1), with a protective effect (meta: *p* = 1 × 10^−5^; Z = −4.4; Figure 2). Intronic variants detected in Nephrocystin 4 (NPHP4) and Latent Transforming Growth Factor Beta Binding Protein 4 (LTBP4) were moderately associated with DNAemia in both cohorts. A SNP in NPHP4 was associated with susceptibility to CMV DNAemia, while a SNP in LTBP4 was associated with protection from CMV DNAemia. An intronic variant in PRR5—ARHGAP8 was found only in cohort B, and a SNP upstream of HLA-DRB1 was exclusive to cohort A. A flavin-containing dimethylaniline monooxygenase 9 (FMO9P) splice donor variant in a pseudo-gene was genotyped in both cohorts and narrowly missed the threshold (meta: *p* = 5.1 × 10^−5^; Z = 4). Gene set analysis of the 100 most significant genes in the DNAemia meta-analysis found significant enrichment of genes associated with autoimmune disease of skin and connective tissue (DOID:0060039: FDR = 0.02; TG, DSG1,DST, LAMA3, HLA-DRB1).

### 3.4. CMV DNAemia Copy Number Association Analysis

Copy number variations (CNVs) were called for the participants with DNAemia, using the participants with undetectable CMV DNA as a reference panel. Each CNV was associated with the genes overlapping the locus (see Supplementary Methods S1 and S2). Sixteen genes had copy variants in 10 viremic patients or more, 14 of which were called in participants of both cohorts. In the viremic group, the LCE3B-LCE3C locus had significant copy loss in both cohorts. There were 28 viremic individuals from both cohorts with copy number loss in the LCE3B-LCE3C locus. Another four genes also had copy loss, albeit not as significant (Table 3, Supplementary File S5).

### 3.5. CMV DNAemia Peak Viral Load Association Analysis

In patients with CMV DNAemia, we tested their peak viral load values for genetic associations. Association analysis on viremic participants reduced the sample size (N = 36, 59 viremic participants in cohorts A and B, respectively), resulting in unreliable results for cohort A and underpowered results for cohort B (Supplementary Files S6–S8). Nineteen variants of interest (*p* < 5 × 10^−5^) were associated with peak viral load for cohort B (Table 4), despite the low statistical power.

## 4. Discussion

Identification of host markers associated with CMV reactivation after transplantation may play a key role in the management and therapeutic decisions for transplant recipients. It is well known that D+/R− transplant recipients are at the highest risk for the development of symptomatic CMV disease [27], but there is a wide variation in the clinical manifestations and outcomes within this group. Some patients develop highly symptomatic CMV with high viral loads, end-organ disease, while others have a milder disease or no CMV DNAemia at all. A better understanding of host protective or risk factors could allow for the personalization of CMV prevention strategies, optimize outcomes, and minimize toxicity and cost. Identification of these host factors requires large, well-designed cohorts with a defined phenotype. Here, we studied two cohorts of CMV-seronegative kidney transplant recipients who received kidneys from CMV-seropositive donors and were treated with the same CMV prophylaxis regimen. Their induction and maintenance immunosuppression regimens were overall similar, and the anti-B cell therapy did not appear to be a risk factor for CMV DNAemia.

CMV DNA was quantified in blood for ~12 months follow-up, differentiating between patients with recurrent CMV DNAemia and those with undetectable CMV DNA. We found a few candidate variants that were associated with the risk of CMV DNAemia. Despite the relatively small sample size, our results indicate that multiple common variants (AF > 10%) significantly affected the risk of developing DNAemia in CMV-naive transplant recipients.

Of the variants associated with protection from CMV DNAemia in both cohorts, several could have a pathogenesis role in CMV (Table 2, Figure 1 and Figure 2). These include a missense variant in dynein (DNHD1) and an intron variant in LTB4 which encodes for a protein that binds to transforming growth factor-beta (TGF-β) as it is secreted and targeted to the extracellular matrix.

Dyneins are broadly associated with viral transport and assembly in the host cell [28] and are considered essential for CMV infection [29]. CMV-infected cells abuse Dynein to maintain favorable mTORC1 activity under stress [30]. Dynein is integral to the formation of the assembly complex and the characteristic large, kidney-shaped nucleus in CMV-infected cells [31]. More recently, it was shown that inhibition of Dynein reduced the number of virions transported to the nucleus and protein synthesis of herpes simplex virus type 1 (HSV1) [32].

An intronic variant in LTBP4 was moderately associated with protection from CMV DNAemia in both cohorts (Table 2). HCMV miRNAs produced during latency induced the expression of TGF-β while protecting the infected cell from TGF-β signaling for efficient viral latency [33]. Transforming growth factor-beta 1 (TGF-β1) was originally reported to stimulate CMV replication [34]. The TGF-β1 promoter was then found to be activated independently by the CMV-encoded immediate-early proteins and transactivated early after infection [35]. TGF-β 1 mRNA increased during the early phase of infection. It has been suggested that induction of TGF-β1 by CMV could modify infected cells and systemic immune reactions to benefit virus replication by both inducing CMV replication and downregulating host immune responses. Urinary excretion of TGF-β is reportedly increased in kidney transplant recipients during CMV infection [36]. Persistent CMV infection in kidney allografts was associated with increased expression of TGF-β [37]. Human renal tubular epithelial cells infected with CMV and exposed to TGF-β1 underwent both morphologic and transcriptional changes of epithelial to mesenchymal transition, similar to uninfected renal epithelial cells. Infected cells also activated extracellular latent TGF-β1 [38].

A SNP in the upstream gene of HLA-DRB1 was associated with susceptibility to CMV DNAemia. HLA-DRB1 was found to be a risk allele after bone marrow transplantation, where in contrast to solid organ transplantation, recipient CMV seropositivity is the highest risk for CMV reactivation. HLA-DRB1*09 was associated with an increased incidence of CMV infection and disease in a cohort of 60 allogeneic hematopoietic stem cell transplant (HSCT) recipients [39]. Among kidney transplant recipients, HLA-DQ3 was identified as an independent predictor of CMV infection in 129 CMV D+/R− patients [40].

CNV is a less-studied type of genomic variation, involved in human disease pathogenesis [41,42]. We detected multiple genes that underwent CNV in a sizable share of both cohorts and in a uniform direction (Table 3). The reproducibility and functional annotations of gene CNVs are suggestive of a substantial effect on the degree of CMV DNAemia. Large duplications/deletions of gene clusters affected multiple genes of the same families, including glutathione S-transferase and Late Cornified Envelope Protein 3D (LCE3D, Table 3). Virus-like vesicles induced the expression of a panel of epithelial differentiation genes, especially genes belonging to the epidermal differentiation complex (SPRR2C, SPRR2D, SPRR3, LCE3D, and SCEL) [43]. The LCE gene cluster members LCE3D and LCE3E were downregulated in an EBV-HPV coinfection model of cervical intraepithelial neoplasia, suggesting that coinfection of EBV and HPV increased the effect of HPV on epithelial differentiation and development [44].

LCE1 genes, located in the LCE gene clusters encoding multiple well-conserved stratum corneum proteins, are reported downstream targets of p53 and regulate protein arginine methyltransferase 5 (PRMT5) activity [45].

Lastly, variants of interest were identified which may be associated with higher peak CMV viral load, although a larger sample size would be needed to confirm these findings (Table 4). Insofar as the magnitude of the CMV peak viral load often reflects the severity of CMV disease, further investigation of these candidate variants would be of interest. NLRC5 is reportedly upregulated in CMV-infected human fibroblasts and plays a role in the JAK/STAT-mediated autocrine signaling loop involving IFN-gamma. Overexpression of NLRC5 protein resulted in activation of the IFN-responsive regulatory promoter elements, IFN-gamma activation sequence, and IFN-specific response element and upregulation of antiviral target genes (e.g., IFN-alpha, OAS1, and PRKRIR) [46].

Our study includes several limitations. The association analysis was underpowered because of the small cohorts. Antiviral immunity is a highly complex, polygenic trait, often studied using cohorts of several thousand individuals [47,48]. The small size of our cohort limits our results to common alleles with relatively high effect sizes and under-estimated significance. The need for larger cohorts is also supported by a study of genetic variants associated with CMV infections after hematopoietic stem cell transplantation [49], showing that most genomic variants previously associated with CMV phenotypes did modify the risk for CMV reactivation or disease after transplantation.

Another limitation is the mostly mono-ethnic composition of our cohort. While the significance of host ethnicity in CMV infection is yet to be determined [50], it is a highly significant factor in the susceptibility and severity of many viral infections [51,52,53]. Ethnic differences may lead to differences in disease severity on a national level [54] or alter the effect of common variants depending on race [55]. Additional advantages include the discovery of rare ethnicity-specific variants [56] and improving the specificity of causal variant detection [57].

Finally, the use of two different sets of exome target sets reduced reproducibility due to lack of overlap. An additional disadvantage of exome sequencing is the decreased sensitivity and accuracy of copy number variant calling [58].

Summarized, despite several limitations, our study provides new insights into complex genetic variants that may play a role in CMV reactivation following kidney transplantation. Larger cohorts as well as future biological systems should validate those markers for future clinical use.

## Figures and Tables

**Table 1:** Descriptive statistics of the cohorts analyzed in the study.

Cohort	A	B	Total
Participants (%)	72 (36.3%)	126 (63.6%)	198 (100%)
Male (%)	41 (56.9%)	75 (59.5%)	116 (58.5%)
CMV DNAemia (%)	36 (50%)	60 (47.6%)	96 (48.4%)
Caucasian (%)	54 (75%)	126 (100%)	180 (90.9%)
Age at time of transplant (Mean, SE)	54.5 (14)	51.2 (1.4)	51.2 (1.4)
Peak viral load (Mean, SE)	2.4 × 10^6^ (1.4 × 10^6^)	6.5 × 10^5^ (2.6 × 10^5^)	1.3 × 10^6^ (5.5 × 10^6^)

**Table 2:** The top 10 variants most significantly associated with CMV DNAemia or protection from DNAemia in a meta-analysis of cohorts A and B. P and beta values are presented for both cohorts, with meta-analysis z-score and *p*-value added. The beta values represent the magnitude and direction of the effect each variant has on the DNAemia outcome. A negative beta value indicates protection, while positive beta values indicate susceptibility to CMV DNAemia.

Variant				*p*-Value			Beta		
ID	GENE	EFFECT	CADD	Meta	A	B	Z-Meta	A	B
11_6569896_G_A	DNHD1	Frameshift, missense	23.5	1.00 × 10^−5^	1.20 × 10^−2^	4.90 × 10^−4^	−4.41	−0.25	−0.28
1_5937391_C_T	NPHP4	Intron	0.184	2.30 × 10^−5^	5.40 × 10^−3^	1.10 × 10^−3^	4.23	0.3	0.24
19_41119273_G_C	LTBP4	Intron	6.661	2.80 × 10^−5^	3.20 × 10^−3^	1.70 × 10^−3^	−4.19	−0.26	−0.24
22_45205158_G_GT	PRR5—ARHGAP8	Intron	2.397	2.80 × 10^−5^	N/A	4.70 × 10^−5^	4.19	N/A	0.38
6_32557621_T_G	HLA-DRB1	Upstream	N/A	4.60 × 10^−5^	4.60 × 10^−5^	N/A	4.07	0.51	N/A
1_166591271_T_C	FMO9P	Splice donor and intron	23.2	5.20 × 10^−5^	9.60 × 10^−3^	1.30 × 10^−3^	4.05	0.34	0.25
21_27840567_C_T	CYYR1	3′ prime UTR	7.693	6.20 × 10^−5^	6.20 × 10^−5^	N/A	−4.01	−0.58	NA
8_126085586_G_A	WASHC5	Intron	1.184	6.40 × 10^−5^	5.90 × 10^−3^	2.70 × 10^−3^	−4	−0.42	−0.25
8_126068873_T_G	WASHC5	Intron	0.375	8.50 × 10^−5^	2.40 × 10^−2^	1.10 × 10^−3^	−3.93	−0.43	−0.36
10_12195881_G_A	SEC61A2	Intron	3.243	8.90 × 10^−5^	8.90 × 10^−5^	N/A	−3.92	−0.37	N/A

**Table 3:** Genes with significant copy number variations in viremic participants compared to the aviremic group. The number of participants with copy gain or loss for a specific gene is shown for each cohort. The adjusted significance (FDR) of the binomial test for each gene is shown.

SYMBOL	A_Loss	B_Loss	A_Gain	B_Gain	A	B	Loss	Gain	Overall	FDR
LCE3B	12	16	0	0	12	16	28	0	28	2.51 × 10^−6^
LCE3C	13	15	0	0	13	15	28	0	28	2.51 × 10^−6^
TAS2R43	5	13	0	0	5	13	18	0	18	1.71 × 10^−3^
GSTM1	17	0	0	0	17	0	17	0	17	2.06 × 10^−3^
GSTM2	17	0	0	0	17	0	17	0	17	2.06 × 10^−3^
AHNAK2	9	3	0	0	9	3	12	0	12	2.99 × 10^−2^

**Table 4:** Variants of interest associated with peak viral load in CMV DNAemia in cohort B.

ID	GENE	EFFECT	*p*-Value	Beta	CADD
13_23808782_T_C	SGCG	Frameshift and missense	1.10 × 10^−5^	5.21	5.754
11_133788869_A_G	IGSF9B	Intron	1.80 × 10^−5^	−3.01	0.173
17_39394962_C_T	KRTAP9-8	3_prime_UTR|intron	2.00 × 10^−5^	2.86	3.321
2_112939548_T_C	FBLN7	Intron	2.10 × 10^−5^	5.07	6.004
2_112940578_C_T	FBLN7	Intron	2.10 × 10^−5^	5.07	1.94
6_36292007_G_A	BNIP5	Intron	2.10 × 10^−5^	4.14	0.052
16_57068107_C_T	NLRC5	Frameshift and missense	2.60 × 10^−5^	−4.42	1.901
16_57071209_T_C	NLRC5	Intron	3.40 × 10^−5^	−3.92	1.492
16_57071226_TCC_T	NLRC5	Intron	3.40 × 10^−5^	−3.92	4.507
16_57071236_C_T	NLRC5	Intron	3.40 × 10^−5^	−3.92	4.909
15_59499179_G_A	LDHAL6B|MYO1E	Frameshift and missense	3.50 × 10^−5^	2.86	8.694
15_59500116_T_C	LDHAL6B|MYO1E	frameshift and missense	3.50 × 10^−5^	2.86	23.1
16_57075406_T_C	NLRC5	Frameshift and missense	3.60 × 10^−5^	−4.48	8.512
16_57076018_G_C	NLRC5	Intron	3.60 × 10^−5^	−4.48	0.213
16_57077523_G_A	NLRC5	Splice region and intron	3.60 × 10^−5^	−4.48	2.641
16_57077581_C_T	NLRC5	Intron	3.60 × 10^−5^	−4.48	0.278
16_57060213_C_T	NLRC5	Frameshift and missense	3.60 × 10^−5^	−4.48	3.843
16_57060340_T_C	NLRC5	Frameshift and missense	3.60 × 10^−5^	−4.48	5.063
16_57060353_T_C	NLRC5	Frameshift and missense	3.60 × 10^−5^	−4.48	0.581

## Data Availability

Data for individual participants is protected under patient confidentiality laws and cannot be shared. See Supplementary Materials section for shared top results.

## Supplementary Materials

The following supporting information can be downloaded at: https://www.mdpi.com/article/10.3390/v15112227/s1, Results and summary statistics for all tests described in this study are deposited at https://zenodo.org/record/8243954. Summary statistics for association tests contain only the 10,000 most significant results. Figure S1A,B: PCA plots of cohort A genotypes, colored according to racial assignment (A) or CMV DNAemia status (B) and shaped according to sex; Figure S2A,B: PCA plots of cohort A genotypes before (A) and after removing outliers (B,C), colored according to racial assignment (A,B) or CMV DNAemia status (C) and shaped according to sex. Supplementary File S1: “variant_calling_summary_stats.xlsx” contains summary statistics for all variants called in cohorts A and B. Supplementary File S2: “dnaemia.cohort1.assoc.txt” genomic associations with DNAemia in Cohort A. Supplementary File S3: “dnaemia.cohort2.assoc.txt” genomic associations with DNAemia in Cohort B. Supplementary File S4: “dnaemia.meta_assoc.txt” genomic meta-associations with DNAemia in Cohorts A and B. Supplementary File S5: “cnv.results.tsv” contains gene-level results associating copy number variants with DNAemia status. Supplementary File S6: “peak_viral_load.cohort1.assoc.txt” genomic associations with peak viral load in Cohort A. Supplementary File S7: “peak_viral_load.cohort2.assoc.txt” genomic associations with peak viral load in Cohort B. Supplementary File S8: “peak_viral_load.meta_assoc.txt” genomic meta-associations with peak viral load in Cohorts A and B. Supplementary Methods S1 and S2: Extended descriptions of the methodology used for cohort A and B, respectively. References [22,59,60,61,62,63,64,65,66,67,68,69] are cited in the Supplementary Methods S1 and S2.
