# Supplementary material for: Genomic Markers Associated with Cytomegalovirus DNAemia in Kidney Transplant Recipients
Source: Viruses. 2023 Nov 8;15(11):2227. doi: 10.3390/v15112227 (PMC10674338; doi:10.3390/v15112227)
Supplement: Supplementary file 1 [file viruses-15-02227-s001.zip › Supplementry methods S2.pdf]

General Methods Summary:
H_Boger_CMV_viremia_SeqWholeExome_210601_1

Sample QC:
Lunatic runs and volume checks are done upon sample receipt at CIDR to confirm adequate quantity and quality of genomic DNA. In addition, samples were processed with an InfiniumQCArray-24v1-0 array to confirm gender, identify unexpected duplicates and relatedness, confirm study duplicates and relatedness, provide sample performance information and sample identity confirmation against the sequencing data. Problems are noted in the problems report (Sample_Info directory).

Exome Capture:
Twist Human Core Exome plus CIDR Custom was used. Lab processing details in next section.

Library Preparation, Enrichment:

LowInputTwist
A low input library prep protocol developed at CIDR (Marosy et al) was performed. Libraries are prepared from 50ng of genomic DNA, sheared for 80s using the Covaris E220 instrument (Covaris). The Kapa Hyper prep kit is used to process the sheared DNA into amplified dual-indexed adapter ligated fragments. All processing was done in 96 well plate formats using robotics (Beckman FXp, Perkin Elmer Janus, Agilent Bravo, Beckman NX). 'With Bead' clean ups were used following shearing and adapter ligation. Amplified libraries were pooled (8-plex, 187.5ng/library) prior to enrichment following the Twist protocol (24 hour hybridization) except for the use of alternate blockers (IDT) and Cot-1 (Invitrogen). Post-enrichment PCR was performed using the Kapa HiFi enzyme according to the Twist protocol, with the adjustment of PCR cycles

Sequencing:
Libraries were sequenced on the NovaSeq 6000 platform using 100 bp paired end runs and S4 Reagent Kit and NovaSeq Xp 4-Lane kit or standard workflow.

Primary Analysis:
Intensity analysis and base calling were performed through the Illumina Real Time Analysis (RTA) software (version 3.4.4). Basecall files were demultiplexed from a binary format (BCL) to single sample fastq files using Illumina's bcl2fastq v2.20.0.422 demultiplexer.

Secondary Analysis:
Fastq files were aligned with BWA mem (Li H. 2013) version 0.7.15 to the 1000 genomes phase 2 (GRCh37) human genome reference. Duplicate molecules were flagged with Picard version 2.17.0. Base call quality score recalibration and binning (2,10,20,30) were performed using the Genome Analysis Toolkit (GATK) (McKenna et al., 2010) version v4.0.1.1. Cram files were generated using SAMTools version 1.7. GATK's reference confidence model workflow was used to perform joint sample genotyping using GATK version 3.7. Briefly this workflow entails; 1) Producing a gVCF (genomic VCF) for each sample individually using HaplotypeCaller (--emitRefConfidence GVCF) and --max_alternate_alleles was set to 3 for all bait intervals to generate likelihoods that the sites are homozygote reference or not 2) Joint genotyping the single sample gVCFs together with GenotypeGVCFs to produce a multi-sample VCF file.

Variant filtering was done using the Variant Quality Score Recalibration (VQSR) method (DePristo et al., 2011). For SNVs, the annotations of MQRankSum, QD, FS, ReadPosRankSum, MQ and SOR were used in the adaptive error model. HapMap3.3, Omni2.5 and 1000G phase high confidence snp calls were used as training sites with HapMap3.3 and Omni2.5 used as the truth set. SNVs were filtered to obtain all variants up to the 99.5th percentile of truth sites (0.5% false negative rate). For indels, the annotations of FS, ReadPosRankSum, MQRankSum, QD and SOR were used in the adaptive error model (4 max Gaussians allowed). A set of curated indels obtained from the GATK resource bundle (Mills_and_1000G_gold_standard.indels.b37.vcf) were used as training and truth sites. Indels were filtered to obtain all variants up to the 99th percentile of truth sites (1% false negative rate).

An additional/optional VCF file was created where genotypes for biallelic SNPs were further refined using CalculateGenotypePosteriors using allele frequency information from 1000 genomes phase 3 data (ALL.wgs.phase3_shapeit2_mvncall_integrated_v5.20130502.sites.vcf) as well as Exome Aggregation Consortium data (ExAC.r0.3.sites.vep.vcf). (Lek, M 2016)

Variant Annotation:
All variants in the final multi-sample VCF file were annotated using Annovar (version 2013_02_21) against a variety of data sources including gene annotation, function prediction and frequency information (see dictionary file).

Summary Statistics:
Summary statistics (for SNVs and INDELs) on the multi-sample .vcf file were calculated for each variant (both PASS and FAIL) including counts and frequencies of alleles and genotypes, missing rates, overall quality scores, and mean depth.

References:

1000 Genomes Project Consortium, Abecasis, G. R., Auton, A., Brooks, L. D., DePristo, M. A., Durbin, R. M., McVean, G. A. (2012). An integrated map of genetic variation from 1,092 human genomes. *Nature, 491*(7422), 56-65. 10.1038/nature11632 [doi]

DePristo, M. A., Banks, E., Poplin, R., Garimella, K. V., Maguire, J. R., Hartl, C., Daly, M. J. (2011). A framework for variation discovery and genotyping using next-generation DNA sequencing data. *Nature Genetics, 43*(5), 491-498. 10.1038/ng.806 [doi]

Fisher, S., Barry, A., Abreu, J., Minie, B., Nolan, J., Delorey, T. M., Nusbaum, C. (2011). A scalable, fully automated process for construction of sequence-ready human exome targeted capture libraries. *Genome Biology, 12*(1), R1-2011-12-1-r1. Epub 2011 Jan 4. 10.1186/gb-2011-12-1-r1 [doi]

Hsi-Yang Fritz M, Leinonen R, Cochrane G, Birney E. (2011) Efficient storage of high throughput DNA sequencing data using reference-based compression. *Genome Research, 21(5),* 734-740. 10.1101/gr.114819.110 [doi]

Lek, Monkol, et al. (2016) Analysis of protein-coding genetic variation in 60,706 humans. *Nature*, 536(7616), 285-291. 10.1038/nature19057 [doi]

Li H. (2013). Aligning sequence reads, clone sequences and assembly contigs with BWA-MEM. *ArXiv:1303.3997v1 [q-Bio.GN],*

McKenna, A., Hanna, M., Banks, E., Sivachenko, A., Cibulskis, K., Kernytsky, A., DePristo, M. A. (2010). The genome analysis toolkit: A MapReduce framework for analyzing next-generation DNA sequencing data. *Genome Research, 20*(9), 1297-1303. 10.1101/gr.107524.110 [doi]

Van der Auwera, G. A., Carneiro, M. O., Hartl, C., Poplin, R., Del Angel, G., Levy-Moonshine, A., DePristo, M. A. (2013). From FastQ data to high confidence variant calls: The genome analysis toolkit best practices pipeline. *Current Protocols in Bioinformatics / Editoral Board, Andreas D.Baxevanis ...[Et Al.], 11*(1110), 11.10.1-11.10.33. 10.1002/0471250953.bi1110s43 [doi] Wang, K., Li, M., & Hakonarson, H. (2010). ANNOVAR: Functional annotation of genetic variants from high-throughput sequencing data. *Nucleic Acids Research, 38*(16), e164. 10.1093/nar/gkq603 [doi]

Wang, K., Li, M., & Hakonarson, H. (2010). ANNOVAR: Functional annotation of genetic variants from high-throughput sequencing data. *Nucleic Acids Research, 38*(16), e164. 10.1093/nar/gkq603 [doi]
