# Supplementary figures and images for: Genomic Markers Associated with Cytomegalovirus DNAemia in Kidney Transplant Recipients
Source: Viruses. 2023 Nov 8;15(11):2227. doi: 10.3390/v15112227 (PMC10674338; doi:10.3390/v15112227)

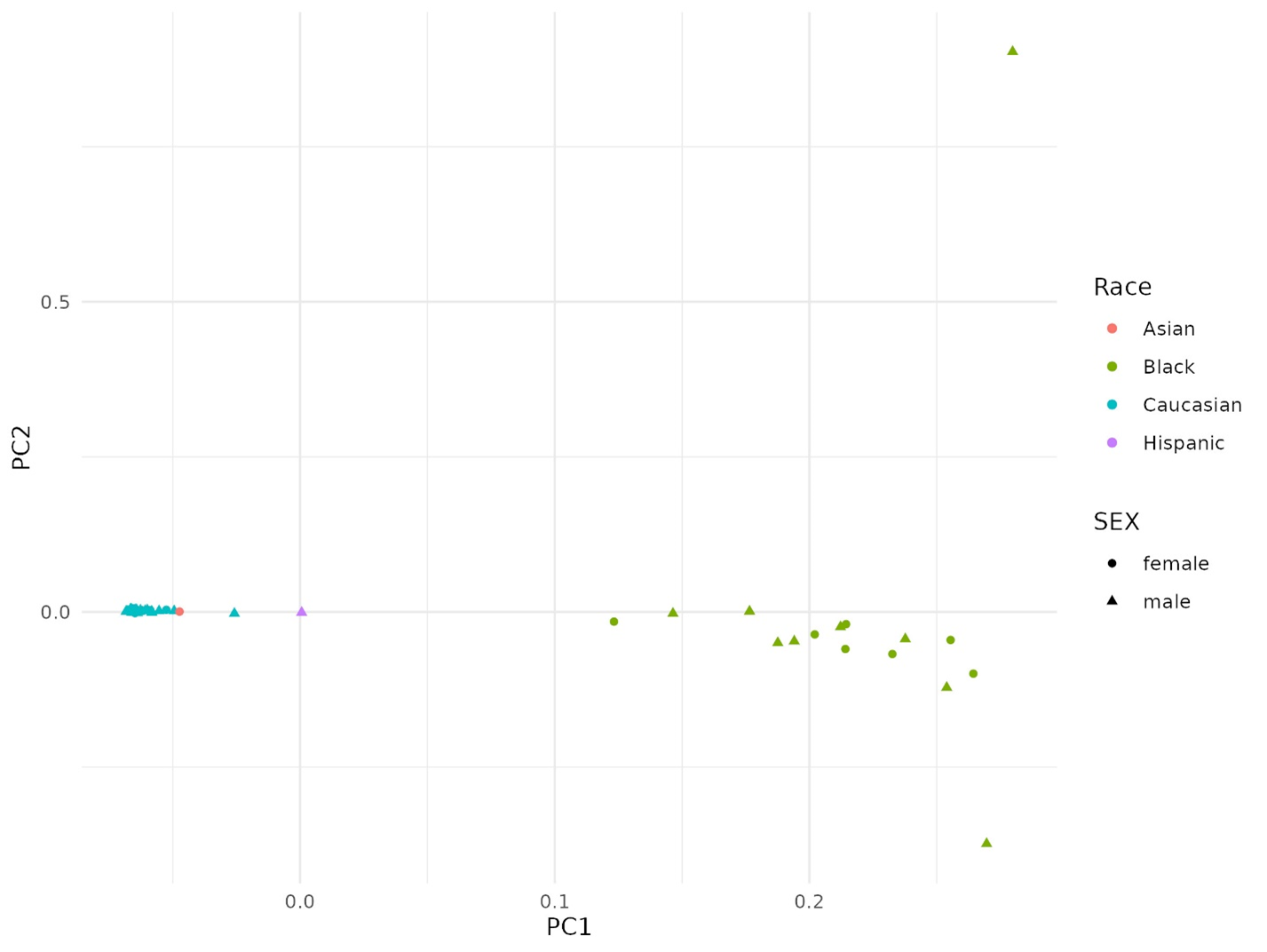

Supplement: Supplementary file 1 [file viruses-15-02227-s001.zip › figureS1A_100523.tif]

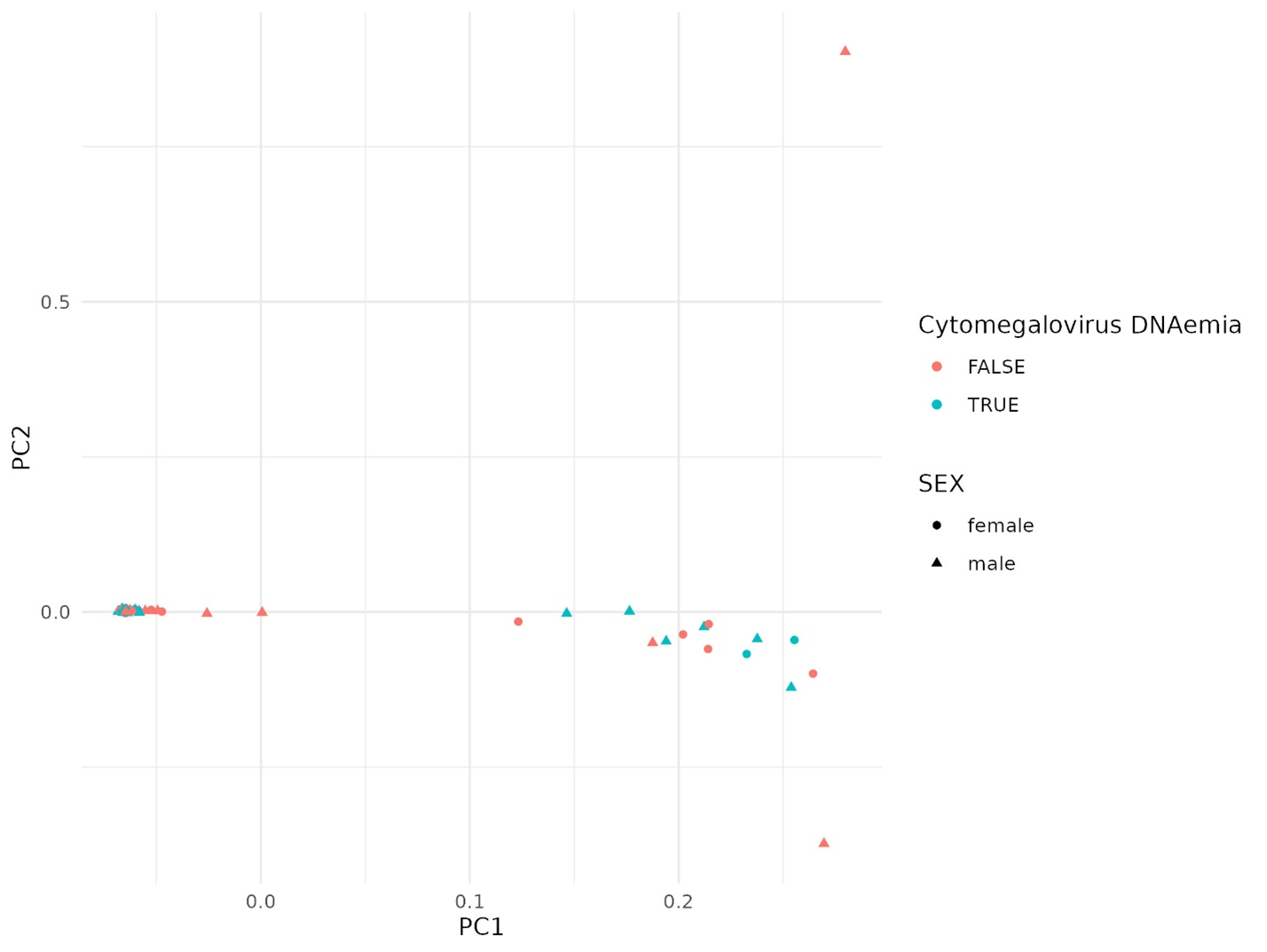

Supplement: Supplementary file 1 [file viruses-15-02227-s001.zip › figureS1B_100523.tif]

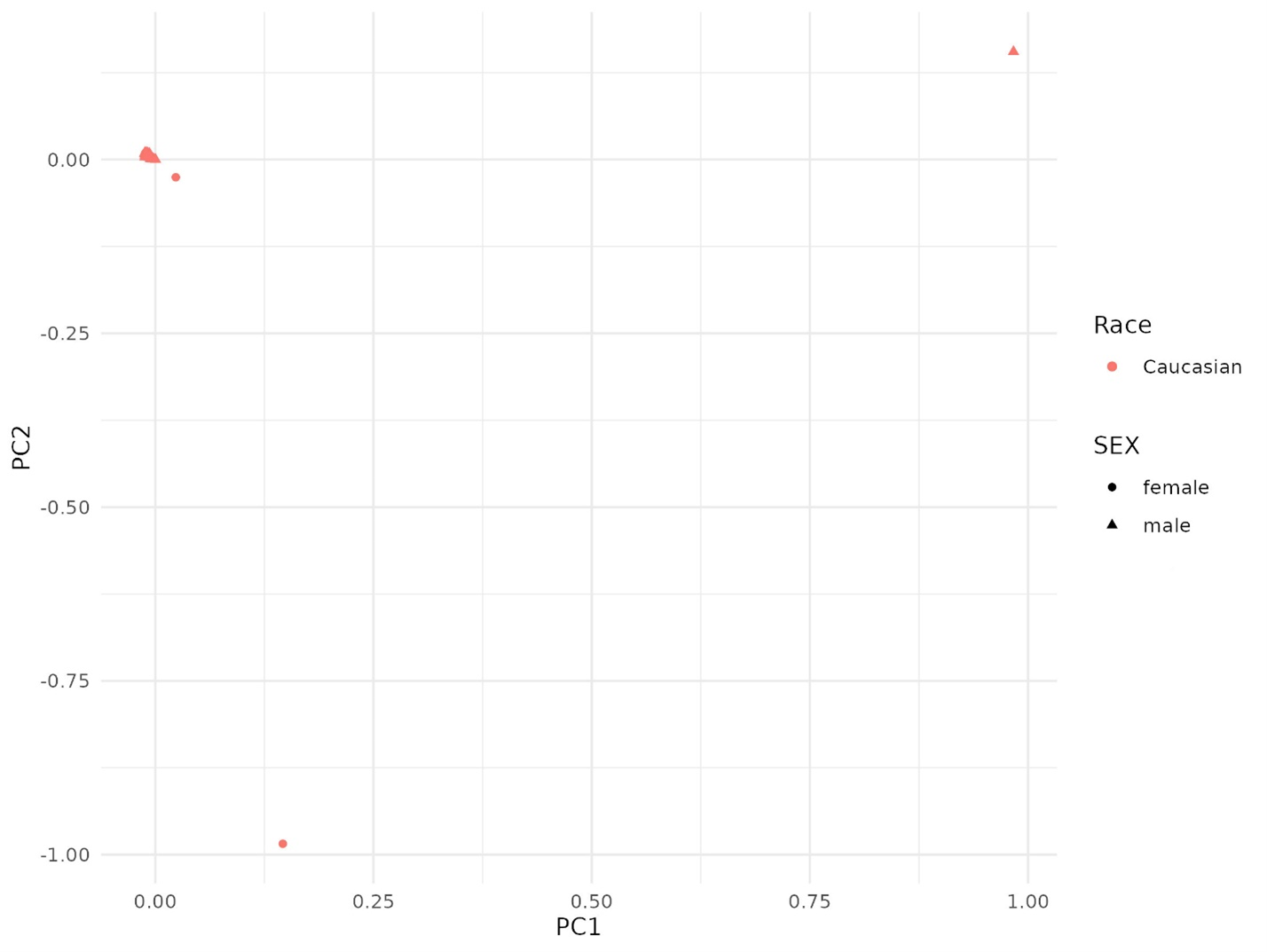

Supplement: Supplementary file 1 [file viruses-15-02227-s001.zip › figureS2A_100523.tif]

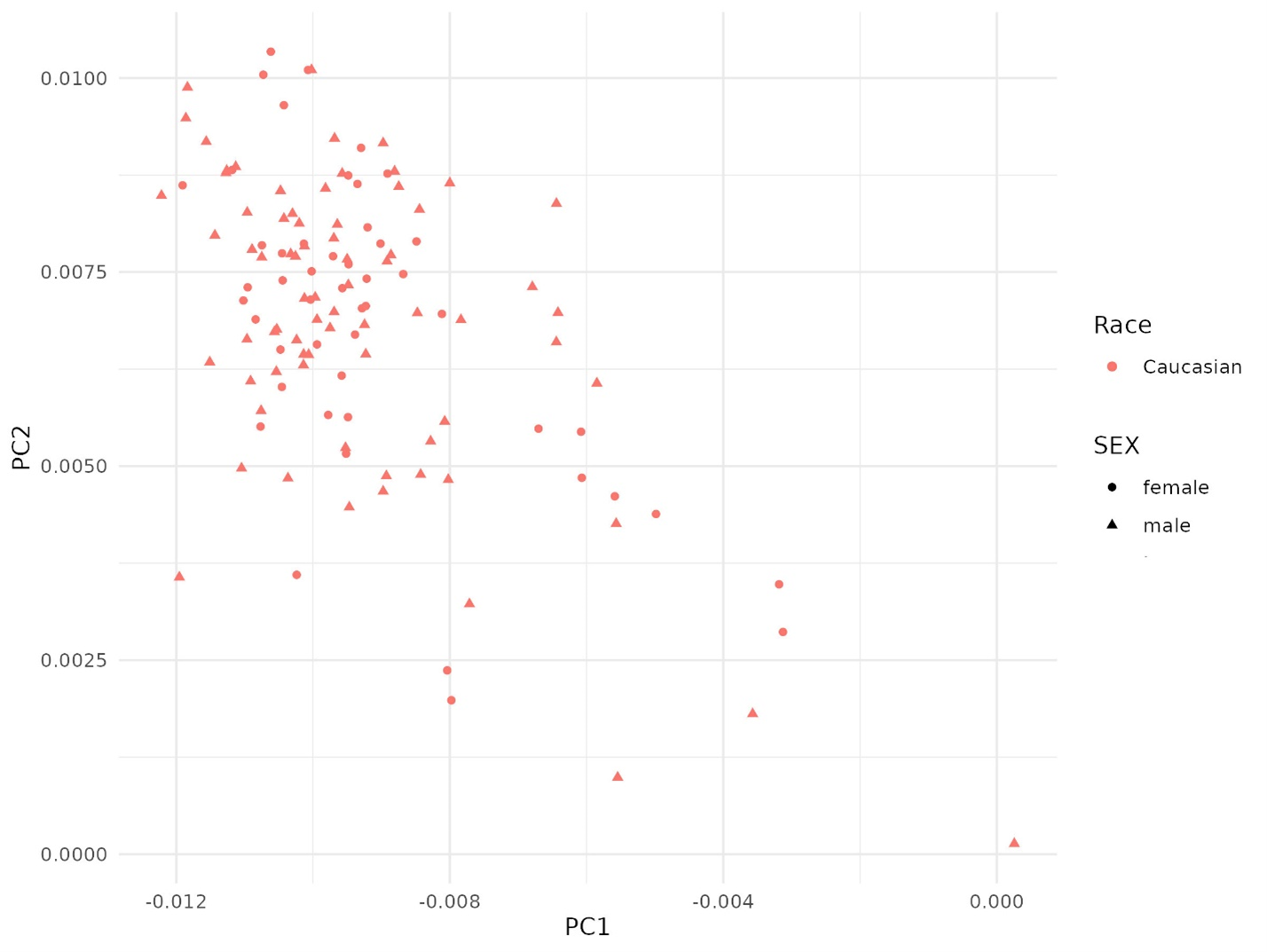

Supplement: Supplementary file 1 [file viruses-15-02227-s001.zip › figureS2B_100523.tif]

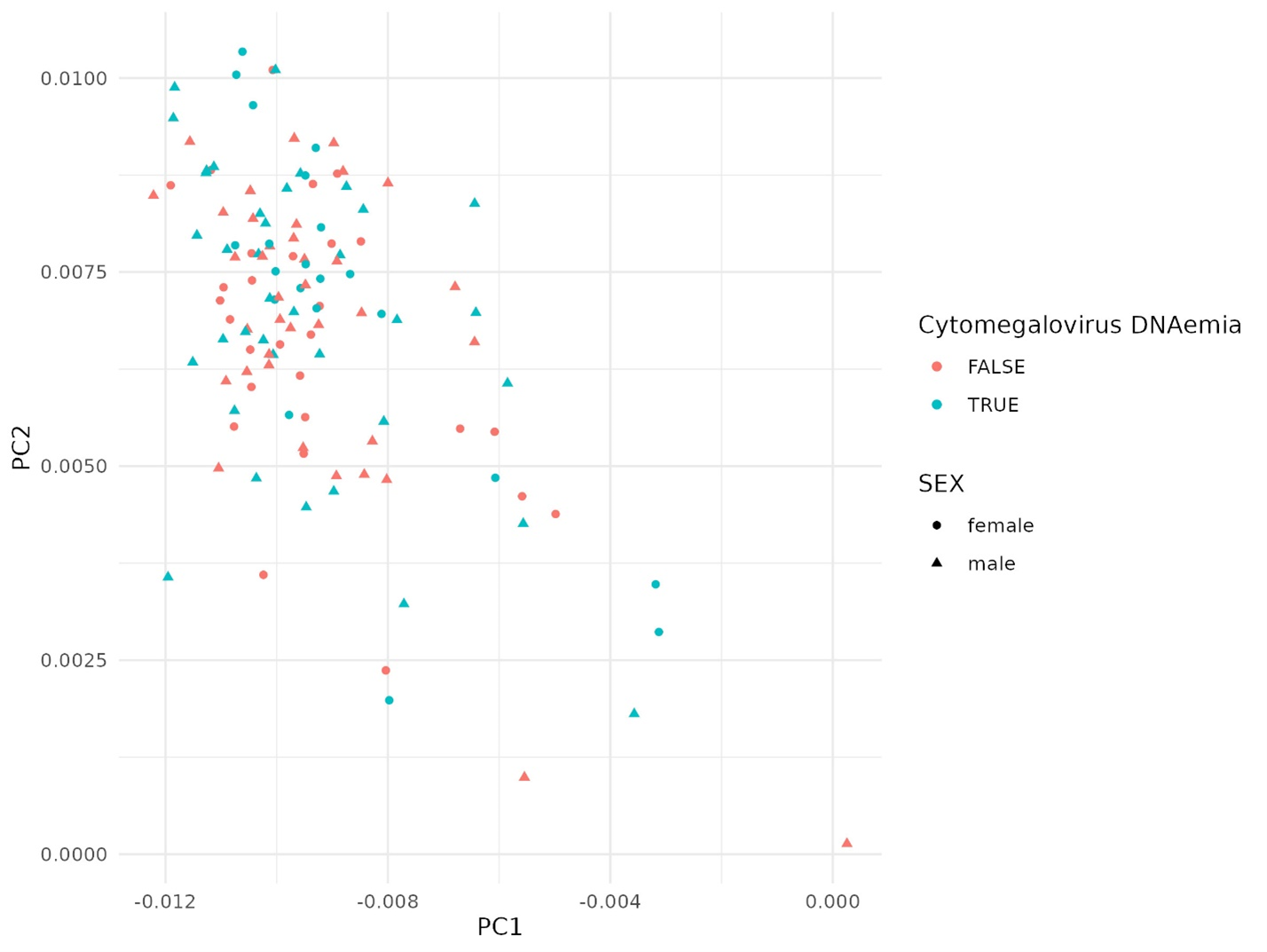

Supplement: Supplementary file 1 [file viruses-15-02227-s001.zip › figureS2C_100523.tif]
